# How medical students cope with stress: a cross-sectional look at strategies and their sociodemographic antecedents

**DOI:** 10.1186/s12909-021-02734-4

**Published:** 2021-05-25

**Authors:** Adam Neufeld, Greg Malin

**Affiliations:** 1 Department of Academic Family Medicine, Cumming School of Medicine, University of Calgary, 3330 Hospital Drive NW, Calgary, AB T2N 4N1 Canada; 2 Department of Academic Family Medicine, College of Medicine, University of Saskatchewan, 107 Wiggins Road, Saskatoon, SK S7N 5E5 Canada

**Keywords:** Medical student, Coping, Stress, Gender, Year

## Abstract

**Background:**

Medical training can be highly stressful for students and negatively impact their mental health. Important to this matter are the types of coping strategies (and their antecedents) medical students use, which are only characterized to a limited extent. A better understanding of these phenomena can shed additional light on ways to support the health and well-being of medical students. Accordingly, we sought to determine medical students’ use of various coping reactions to stress and how their gender and year of study influence those behaviours.

**Methods:**

A total of 400 University of Saskatchewan medical students were invited to complete an online survey. Using the Brief COPE inventory, we assessed students’ reported use of various adaptive and maladaptive coping strategies. Descriptive and comparative statistics were performed, including multivariate analysis of variance, to explore how gender and year influenced coping strategies.

**Results:**

The participation rate was 49% (47% males and 53% females). Overall, the students’ coping strategies were mostly adaptive, albeit with a few exceptions. Females used more behavioural disengagement, while males used less emotional and instrumental support. Additionally, third years used more denial to cope with stress than students in any other year.

**Conclusions:**

While few studies report significant sociodemographic effects on medical student coping, our findings raise the possibility that males and females do engage in different coping strategies in medical school, and that the clinical learning environment in third year may provoke more dysfunctional coping, compared to pre-clinical stages of training. Potential explanations and implications of these results are discussed.

## Background

During medical school, students’ mental health is known to be at significant risk [1, 2]. Hence, there is an active search to better understand and promote their health and well-being. While the prevalence of distress has been established among medical students [1–3], less is known about the types of coping behaviours they engage in to mitigate distress throughout their medical education [4]. A better grasp of this is important, because how medical students cope not only predicts their mental health [5] and academic performance [6], but the quality of patient care they go on to deliver [7, 8]. In view of this, the present study adds to the literature by exploring the extent that students use various coping strategies in medical school and whether those strategies vary as a function of gender and year of training. We start by examining the extant literature on stress and coping, both in and outside of medical education. We then show how often medical students use various coping strategies to deal with stress, and to what extent their gender and year of training explain the observed variance in those outcomes. A discussion of results follows, including potential implications in medical education. Our hope is that this research may advance our understanding of student stress and coping in medical school, as well as guide the optimization of supports for medical students, in ways that maximize their health and well-being.

### Coping

Coping has been extensively studied and conceptualized in various ways in the literature [9, 10]. Here we define coping as a conscious volitional effort to regulate one’s emotions, thoughts, and behaviours, in response to stress [11]. While it is well-established that coping can be categorized into two main types: problem-focused and emotion-focused [12], there is also a consensus of a second-order dimension of coping—namely, adaptive (i.e., “active”) and maladaptive (i.e., “passive” or “avoidant”) [13–15]. Adaptive coping involves flexible approaches to solving problems and/or managing their related emotions (e.g., through strategizing, reappraisal, and emotional regulation and expression) [10], whereas maladaptive coping involves behaviours that are less constructive and fruitful (e.g., ruminating, venting, confrontation) and avoidant (e.g., abandonment, social isolation, and inhibiting or suppressing one’s emotions) [16, 17]. These dimensions of coping, which provide a framework to help contextualize the adaptivity of strategies used by medical students, are categorized and defined below (see Table 1).

**Table 1:** Categorization of Brief COPE strategies (adapted from Carver [14] and Rice et al. [18])

**Adaptive**	**Emotion-focused strategies**	
	*Acceptance*	Accepting the reality of a stressor and learning to live with it
	*Emotional support*	Getting moral support from others
	*Positive reframing*	Trying to view stressors in a different, positive light
	*Humour*	Using humour and making fun of the stressor
	*Religion*	Trying to find comfort in one’s religion or spiritual beliefs
	***Problem-focused strategies***	
	*Active coping*	Concentrating efforts on trying to make the situation better
	*Planning*	Thinking hard about strategies to manage the stressor
	*Instrumental support*	Trying to get advice or help from other people on what to do
**Maladaptive**	***Passive and/or avoidant strategies***	
	*Denial*	Refusing to believe or pushing the reality of a stressor away
	*Self-distraction*	Focusing on other things to take one’s mind off the stressor
	*Substance use*	Using alcohol or drugs to feel better and deal with the stress or
	*Behavioural disengagement*	Giving up trying to manage or cope with the stress or
	*Venting*	Focusing on and verbalizing negative feelings
	*Self-blame*	Criticizing and blaming oneself for what happened

### Stress and coping in medical school

Medical students deal with many types of stressors in medical school: intense academic demands and workloads, challenging curricular aspects and learning environments, personal life events, and psychological pressures that are difficult to cope with [19–22]. In fact, in a recent study concerning medical student resilience and the roles of coping styles and social support, it was found that 49% met criteria for burnout, 17% had moderate to severe depression, and 23% responded that developing depression was due to their inability to cope [23]. Studies like these highlight how important healthy coping behaviours are for medical students, in being able to overcome the stressors they face in medical school [3, 24].

Research in medical education supports the proposed relations between adaptive and maladaptive coping styles, with positive and negative mental health outcomes, respectively. For example, more active (e.g., seeking social support) versus passive and avoidant (e.g., drinking alcohol) coping strategies have be shown to be key mediators of medical students’ burnout during medical school, as measured by the Maslach Burnout Inventory (Student Version) [25]. Other studies are complimentary, showing that when medical students utilize more adaptive coping behaviours, it relates to lower levels of distress and higher resilience [4, 23]. Nonetheless, sociodemographic antecedents (e.g., year of study, gender, ethnicity) of medical students’ coping behaviours are not well characterized and there has been a call for more research on the specific types of coping strategies they use, in order to buffer stress [4, 26].

### Gender differences in coping

Within the general population, studies have reported significant gender differences in coping behaviours—for instance, that females tend to experience more chronic stress and consider stressors as more threatening than males [27–29]. Studies also suggest that females vent more emotions and rely on more social support to cope with stress (e.g., instrumental and emotional support), while males may use more passive and/or avoidant methods of coping, like alcohol or drugs [30, 31]. Other research has found that females tend to use more maladaptive coping strategies than males, such as self-distraction, denial, and behavioural disengagement [10]. Interestingly, these differences have largely been attributed to social norms and gendered constructs, rather than actual sex differences [32, 33]. Hence, workplace climates can dramatically shape coping behaviours, based on how accepted and reinforced those “masculine” (i.e., agentic and instrumental) or “feminine” (i.e., communal and emotional) traits tend to be [34]. Indeed, the extant literature on social roles and gender differences in health, stress, and coping, supports this notion [27, 35–37].

Despite a consensus of what generally constitutes adaptive vs. maladaptive coping reactions, and that gender roles play a part, the literature remains mixed regarding specific gender differences in coping. It is also worth noting that most studies have looked at gender as a binary construct and there are really no studies that have taken into consideration non-binary gender classifications, in terms of coping. While some traits that are considered “masculine” have been associated with primarily adaptive coping strategies [38], others have reported positive and negative associations (e.g., with seeking social support) [38, 39]. Further, while females are found to experience higher distress than males, studies have also linked masculinity to distress, through unhealthy stress appraisals and disengagement coping [40]. Whether these delineations reflect unique sample characteristics or under-reporting on surveys remains unclear. Some have suggested that it more likely reflects an equalization of gender roles, due to increasing female representation in the workplace [29, 41]. Thus, an evolution in gender role attitudes and environments may also explain gender-related coping differences, particularly among physicians [42]. This leads us to consider whether gender differences in stress and coping might too be evolving in medical education—where more females are now enrolled than males for the first time in recent history [43].

### Gender and medical student coping

In keeping with the broader literature, gender differences in coping strategies among medical students are also somewhat enigmatic. For instance, a recent study looking at latent profiles of coping among medical students found that gender was not a significant correlate of coping styles [4]. Various other studies are also in agreement with this notion, that coping differences do not differ among male and female medical students [24, 44, 45]. In contrast, however, others have found significant gender differences in coping among medical students—for example, that females preferred to study and sleep, while males preferred to spend time with friends, play sports, or isolate themselves [19]. Interestingly, some studies have also found male medical students to score higher in distress than females, which related to more maladaptive coping strategies like self-blaming, denial, and substance use [46]. This finding is somewhat distinct from other areas in the literature, where females tend to score higher in distress. Since many of these investigations were conducted in Eastern vs. Western nations, further research is required to determine the extent that cultural and environmental factors contribute to how male and female medical students cope.

### Environmental considerations in medical student coping

As mentioned, other important determinants of how medical students cope are curricular and environmental factors [21]. Indeed, there is a growing body of evidence supporting that the further medical students get in their education, the more emotionally taxing it might be. This could be because clinical students encounter situations they will have never experienced before (e.g., patient deaths, delivering bad news, child abuse), or because they may be relatively disconnected from social networks they had in place during pre-clinical years, which served as a buffer for dealing with stress. Alternatively, changes in coping could reflect the uncertain nature of the stressors clinical vs. pre-clinical students face. For instance, after adjusting for gender, ethnicity, and school, one study reported worsening student perceptions of the learning environment in third year of medical school (when clinical rotations start), with some recovery after “match day” in fourth year (when students learn of their residency placements) [47]. These findings correlate with other reports in the literature, showing that medical students in later vs. earlier years of training tend to use more avoidant, maladaptive coping strategies, which tend to emanate when stressors are perceived as uncontrollable [25, 48, 49]. Hence, how students cope likely depends on the unique environments and stressors they face in each year of medical training. While differences in distress are well-documented among medical students in pre-clinical vs. clinical years [3, 50], how their respective coping behaviours might also differ remains relatively unclear.

### Current study

The present study aims to extend prior research by exploring what types of specific coping strategies medical students report using to buffer stress in medical school, and how their gender and year of training influence those coping behaviours. Considering the extant literature on these topics, as well as the uniquely stressful nature of medical school (which includes many challenges that learners may view as more or less controllable) [51, 52], our hypotheses are twofold. First, females will use more maladaptive coping strategies (e.g., venting, behavioural disengagement, and/or self-blame) [10], while males will use fewer adaptive ones (e.g., seeking emotional and instrumental support) [19]. Secondly, medical students in clerkship (third and fourth years) will use more maladaptive coping strategies (e.g., denial, self-distraction, self-blame) compared to those in pre-clerkship (first and second years) [25, 48, 49].

## Methods

### Participants & procedure

A cross-section of 400 medical students, from all 4 years of the medical program at the University of Saskatchewan, were invited to complete an online survey, toward the end of the 2019 school year. The survey included information about the study and measured how frequently students used various coping strategies in medical school (see Measures). Participation was voluntary and responses were anonymous, to maintain confidentiality and minimize response bias.

Of note, the 4-year medical program at the University of Saskatchewan—much like the majority of medical programs in Canada—separates its curriculum into two main components: pre-clerkship (years 1 and 2) and clerkship (years 3 and 4). Students in pre-clerkship learn mostly through classroom-based modules, small group sessions, clinical integration (weekly skills practice and experiential patient encounters), and regular examinations (both written and clinical). In contrast, students in clerkship primarily work in clinical environments (e.g., clinics and hospitals). Duties at this stage tend to provide more independence but include challenging national board examinations, longer, mandatory work hours, and higher patient responsibilities.

### Ethical approval

This research received ethics approval from the University Research Ethics Board. All participants provided informed consent.

### Measures

The survey contained questions related to the medical students’ year of study (1–4) and gender identity (“male,” “female,” or “other”), followed by the Brief COPE inventory, which was oriented to reflect students’ experiences in medical school. Of note, there were no major social events that occurred during the academic year, which we felt could influence results.

#### The Brief COPE (Carver [14])

The Brief COPE is a 28-item scale containing 14 two-item factors. It measures how frequently people use a range of distinct coping reactions to stress (active coping, planning, acceptance, positive reframing, emotional support, instrumental support, humour, religion, denial, venting, self-distraction, behavioural disengagement, and self-blame). Items were presented in a retrospective manner (i.e., looking back throughout the year) and participants indicated how frequently they used each coping strategy on a scale, ranging from 0 (*I haven’t been doing this at all*) to 3 (*I have been doing this a lot*). Thus, for each of the 14 COPE factors, the mean score could range from 0 to 6, as an average of each subscale’s two items. Mean subscale scores were calculated by adding item scores together. As Carver recommends [14], the wording of the scale was modified for this study, to fit the population of students and challenges they face in medical school. For instance, whereas part of the original Brief COPE's stem read, “We want to know to what extent you’ve been doing what the item says,” we added, “…since starting your year in medical school.”

The Brief COPE has been validated in health-related research and its subscales have been shown to have satisfactory reliability (with Cronbach’s alpha’s from 0.50 to 0.90) [14]. Prior exploratory and confirmatory factor analyses support Carver’s proposed 14-factor structure of coping [53, 54], along with its higher order (adaptive and maladaptive) categorization [10, 12, 55–57]. Hence, studies support the usefulness of the situational version of the Brief COPE (used in the present study), for the valid and reliable assessment of the 14 specific coping responses to stressors. Accordingly, we assessed the medical students’ reported use of every strategy, without attempting to create new variables or reduce the data’s dimensionality (i.e., through principal components or factor analysis). Instead, we draw on the supporting literature to help identify which strategies the students report using that might be healthy vs. dysfunctional, in a medical education context.

### Statistical analyses

The software SPSS version 24.0 was used for our statistical analyses. All data were standardized and met the assumptions of normality, linearity, and homoscedasticity. Descriptive statistics determined the students’ mean scores for each coping strategy. Relationships between the coping factors were assessed using Pearson correlations. We then explored whether students’ coping strategies varied as a function of gender and year of study, using one-way multivariate analysis of variance (MANOVA) with post-hoc unpaired *t*-tests (or pairwise comparisons) and 95% confidence intervals, to see where the differences lay. Levene’s test of equal variances and Bonferroni’s *p*-value correction were used where appropriate. Partial eta squared (*η*_*p*_^*2*^) values were provided as effect sizes for the MANOVA (where .01 is considered small, .06 is medium, and .14 is large). Cronbach’s alphas were calculated and ranged from 0.49–0.93, which were deemed to be acceptable based on prior studies using the separate Brief COPE subscales [58–60].

## Results

### Descriptive statistics

The response rate of the students was 214/400 (54%). However, surveys from 17 participants were insufficiently completed and thus were excluded from the analyses. This left a total of *n* = 197 participants (49%)—92 who identified as “male” (47%), 105 as “female” (53%), and none as “other.” The sample consisted of 70 (36%) first years, 57 (29%) second years, 35 (18%) third years, and 35 (18%) fourth years. The mean age was 25.9 years (*SD* = 3.7) with a range of 21–44 years. As seen in Table 2 below, the sample’s Brief COPE subscale scores are listed in descending order of reported use (see Measures for interpretation).

**Table 2:** Brief COPE subscale scores among the medical students

Coping strategy	M (SD)
Active coping	5.99 (1.42)
Emotional support	5.85 (1.69)
Planning	5.82 (1.42)
Acceptance	5.70 (1.37)
Instrumental support	5.64 (1.70)
Positive reframing	5.41 (1.61)
Self-distraction	5.30 (1.53)
Humour	4.88 (1.78)
Self-blame	4.77 (1.66)
Venting	4.35 (1.58)
Religion	3.37 (1.83)
Substance use	3.01 (1.61)
Behavioural disengagement	2.76 (1.10)
Denial	2.36 (0.85)

*M* mean, *SD* standard deviation

As Table 2 illustrates, the Brief COPE subscale scores suggest that the medical students use predominantly adaptive (e.g., active coping, emotional support, positive reframing) rather than maladaptive (e.g., denial, behavioural disengagement, substance use) coping strategies, in response to stressors they face in medical school. Interestingly, however, several strategies that are generally considered maladaptive also appear fairly frequently—for instance, venting, self-blame, and in particular, self-distraction. These patterns informed the next step, which explores how the different coping strategies correlate.

### Variable relationships

Table 3 shows the intercorrelations for all study variables. Overall, the strength of relationships are low to moderate range and are in the expected directions. For instance, coping strategies that are generally considered adaptive (i.e., planning, acceptance, emotional and instrumental support, positive reframing, humour, and religion) positively relate to one another and negatively correlate with coping methods that are typically considered maladaptive (e.g., denial, substance use, behavioural disengagement, self-blame, self-distraction). Interestingly, however, we find other less expected patterns among this sample of medical students—for example, with venting (which positively relates to acceptance, planning, emotional and instrumental support, and humour), and the positive relations between instrumental support and self-blame, humour and substance use.

**Table 3:** Intercorrelations between demographic and coping variables among medical students

	1	2	3	4	5	6	7	8	9	10	11	12	13	14	15	16	17
1. Age	–																
2. Gen	**−**.03	–															
3. Year	.25**	.03	–														
4. D	−.04	.01	.01	–													
5. SU	−.04	−.04	−.06	**.**26**	–												
6. BD	−.05	.19*	.07	.30**	.33**	–											
7. V	.09	.02	.06	.09	.14	.20**	–										
8. SB	.07	.11	−.05	.17*	.21**	.37**	.14	–									
9. SD	−.09	.11	−.04	.07	.11	.07	.13	.20**	–								
10. AC	.05	−.09	−.05	−.08	−.08	−.34**	−.01	−.14	−.12	–							
11. ES	−.12	.28**	−.05	−.03	.13	−.02	.38**	.14	.06	.23**	–						
12. IS	.09	.26**	.06	.01	.07	−.05	.44**	.20**	.10	.20**	.70**	–					
13. PR	−.01	−.05	−.02	−.03	.13	−.29**	.04	−.17*	−.15*	.48**	.29**	.28**	–				
14. P	.15*	−.07	.03	.02	.08	−.25**	.16*	.06	−.09	.62**	.22**	.33**	.51**	–			
15. A	.06	.02	−.02	−.15*	.05	−.10	.19*	−.01	.09	.37**	.21**	.24**	.39**	.40**	–		
16. H	.06	−.09	−.12	−.05	.22**	−.07	.28**	.08	.12	.19**	.18*	.16*	.32**	.29**	.27**	–	
17. R	−.01	−.04	−.01	−.04	−.11	−.06	.12	−.02	−.15*	.18*	.19*	.25**	.19**	.17*	.04	−.07	–

*Gen* gender (1 = male, 2 = female); Year (1 to 4), *D* denial, *SU* substance use, *BD* behavioural disengagement, *V* venting, *SB* self-blame, *SD* self-distraction, *AC* active coping, *ES* emotional support, *IS* instrumental support, *PR* positive reframing, *P* planning, *A* acceptance, *H* humour, *R* religion

* *p* < .05 and ** *p* < .01 (2-tailed)

With respect to demographics, results indicate a weak but significant positive correlation between age and planning. Point biserial correlations also suggest that females use significantly more behavioural disengagement to cope, while males use significantly less instrumental and emotional support to cope. The correlational analyses did not detect a significant relationship between students’ year of training and the types of coping strategies they use.

### Sociodemographic effects on medical student coping

Though no significant correlations were identified between year and coping, our a priori hypotheses were that coping differences would likely be influenced by students’ gender and year of training. Thus, a one-way MANOVA was used to assess the effect of gender and year on each of the Brief COPE subscales. Levene’s tests indicated equal variances for all coping factors across gender and year subgroups (*p*’s > 0.05). Interestingly, the MANOVA found a significant effect of year on denial, *F* (3,181) = 3.16, *p* = 0.03, η_p_^2^ = .05. Follow-up pairwise comparisons revealed that third year students reported the highest use of denial, which differed significantly from fourth years, who reported the lowest use of this strategy (*MD* = .61, *SE* = .21, *p* = .03). Additionally, there was a notable effect of year on behavioural disengagement that approached but did not achieve statistical significance, *F* (3,181) = 2.64, *p* = .051, η_p_^2^ = .04. Follow up pairwise comparisons again confirmed that third years scored highest, this time differing most from first years, who scored lowest (*MD* = .63, *SE* = .24, *p* = .052). The second MANOVA confirmed significant gender effects on several of the medical students’ coping strategies. Specifically, males sought less emotional support, *F* (1, 181) = 16.57, *p* < .001, η_p_^2^ = .08, as well as instrumental support, *F* (1,181) = 13.84, *p* < .001, η_p_^2^ = .07, and females used more behavioural disengagement to cope, *F* (1, 181) = 8.65, *p* = .004, η_p_^2^ = .04. Of note, the effect sizes of students’ year and gender on each coping strategy are considered small to medium.

## Discussion

The present study is among the first to assess how a subset of Canadian medical students cope in response to various stressors in medical school, and how their gender and year of study impact those coping behaviours. As was hypothesized, we found significant associations between both sociodemographic antecedents and the students’ reported coping strategies. Specifically, females reported greater use of behavioural disengagement and males reported less reliance on emotional support and instrumental support. Moreover, we found that third year students reported the most use of denial to cope than all other years. Potential explanations and implications of these findings are discussed, with suggestions for future research below.

### Medical students’ overall coping

As mentioned, the Brief COPE measures fourteen distinct coping responses to stress—some adaptive for health and well-being and others less so [14]. While we cannot comment generally on how students cope with stress, our findings suggest that the coping strategies adopted by the medical students were primarily healthy, rather than dysfunctional (see Table 2). Breaking things down further, this notion is substantiated by the students’ relatively equal use of problem-focused (e.g., planning, active coping, seeking instrumental support) and active, emotion-focused (e.g., seeking emotional support, positive reframing, humour) coping strategies, compared to their less frequent use of passive and avoidant ones (e.g., denial, substance use, behavioural disengagement).

In support of Carver’s 14-factor structure of coping [14], many of the individual coping strategies did not correlate with one another, in the present study. This was to be expected, given that the Brief COPE is comprised of coping reactions that are said to be distinct (i.e., which neither directly relate much, nor oppose each other, but rather co-exist). Hence, they are assessed individually and in relation to each other, and more as a way of identifying overarching patterns, since each coping strategy is broadly understood to be adaptive or maladaptive. In line with this notion, most of the coping behaviours that the medical students reported using which are considered adaptive or maladaptive do intercorrelate, respectively. Accordingly, the number and potentially weak or moderate strength of the significant correlations are not considered a shortcoming, but rather a testament to the importance of studying all 14 strategies, to fully capture how medical students cope with stress.

Interestingly, despite the above findings, several frequently used strategies did emerge that might not be as adaptive—for instance, students’ use of self-blame, self-distraction, and venting. This might be explained by previous studies showing that, while certain coping strategies like planning, active coping, and instrumental support consistently relate to better health outcomes than dysfunctional strategies [4, 14, 61], other coping methods are not always as stable or predictable [13]. A fitting example of this was the medical students’ reported use of venting, which positively related to mostly adaptive coping strategies (e.g., emotional and instrumental support, humour, planning, and acceptance) and not maladaptive ones that it has commonly been associated with (e.g., denial, self-blame, substance use) [14, 62]. This might be explained by medical students associating the humorous telling of frustrating or unusual experiences they encounter in medicine, as venting, which differ from other workplace settings, in the wide range of experiences and novelty they present to early medical learners. In support of this, Park et al. [63] explained that even avoidant and/or emotion-focused coping strategies may become useful—particularly when a stressor is felt to be overwhelming or out of one’s sense of control. Either way, it lends support to the idea that some emotion-focused coping strategies may be more or less adaptive depending on the situation [64, 65]. Though there is little consensus about which coping strategies are in fact most effective, studies do generally support this premise regarding controllability [66, 67].

Another interesting finding was that the medical students’ use of self-blame positively related to them seeking instrumental support. Other studies have also reported this finding in the literature [62]. Hence, while our overall results suggest that medical students wish to cope adaptively with the high demands of medical school, it is possible that they also blame themselves for not always being able to achieve that goal. And, based on our correlational data, they may resort to self-distraction or other means to cope. As others have explained, self-blame may thereby work as a double edged-sword, given it can stimulate active coping behaviours, such as seeking instrumental support, or conversely lead to guilt and feelings of depression [62]. This could be explained by the difference between guilt vs. shame, where guilt is more motivating for corrective action, while shame is more pathogenic as it relates to the self [68]. This link between active coping, self-blaming, and self-distraction in medical school, is a subject for further research.

Put together, the patterns of the above findings align with others in the literature, highlighting specific coping strategies and their frequency of use among medical students [69, 70]. They also suggest that medical students may use more active or problem-focused types of coping (e.g., planning) to deal with stressors that they view as solvable (e.g., studying hard to pass a tough exam) and potentially more passive and/or avoidant methods of coping (e.g., venting, denial, self-distraction, behavioural disengagement) to deal with challenges they feel are uncertain and more daunting (e.g., matching to their residency program of choice). The overlap between coping and resilience is also a consideration here, but one that is beyond the scope of this paper.

### Coping differences by year of training

Of note, the only coping strategy that was found to vary by students’ year of study was denial (refusing to accept or face the reality of a situation), which was highest among third years and lowest among fourth years. While it did not achieve statistical significance, our data also suggest that third years might resort to more behavioural disengagement. As mentioned, coping with these strategies tends to predict distress in the long-term [14]. These patterns, which align with other studies in the medical education literature [49, 70], may reflect the fact that third year represents a very challenging stage for medical students—when they face many more imminent stressors (e.g., more patient care, stressful board examinations, competitive electives, long work hours, sleep deprivation, and higher worry about the future) [48, 51, 71, 72]. Our and others’ findings further suggest that fourth year likely relieves medical students of many of these uncertainties, thereby removing their need to use passive and avoidant coping strategies, such as denial [47].

In terms of why third years may be using more denial to cope, it could be related to feelings of helplessness—for instance, about the uncertainty of securing competitive electives and the process of matching to residency, in the year ahead. Indeed, studies show that when a person feels they have little or no control over the outcome of a situation, they will tend to engage in more avoidant and/or emotional coping styles, as a stress-protective mechanism [12, 56]. It therefore follows that denial might be higher among third year medical students because they perceive less autonomy during this stage of their training—something others have emphasized the importance of for physicians and medical students alike [73–75]. Support for this theory comes in a recent study which showed that, among a four-year sample of medical students, third years reported the highest autonomy frustration and perceived stress compared to the other 3 years [76]. An alternative explanation might be that third years are placed into situations they have simply never encountered before (e.g., dying patients, witnessing or delivering bad news) that may feel surreal and be harder to cope with actively and adaptively at first, compared to fourth years, who have likely experienced, reflected on, and acclimated to these types of challenging encounters. Fourth years may also have less of these experiences to adapt to over their year, compared to new clerkship students. As with venting, self-distraction, and self-blame, more research is needed to determine if and how long it might be before maladaptive coping strategies, such as denial, shift from being stress-protective for medical students to being psychologically harmful.

### Gender differences in coping

With respect to gender differences, another interesting finding of this study was that female medical students reported more use of behavioural disengagement (giving up trying to deal with a situation), while males reported less reliance on emotional support (seeking comfort and understanding from others) and instrumental support (getting advice from others) to cope. Once more, these forms of social support are considered healthy and adaptive, whereas behavioural disengagement tends to predict maladjustment over time [14]. While various studies purport no gender-based coping differences among medical students [24, 44, 45], our findings align with other studies that do, both within and outside of medical education—for instance, that male medical students used more isolating (i.e., less social) coping strategies than females [19], and that females used more behavioural disengagement than males, respectively [10].

As previously mentioned, the literature suggests that any gender differences in coping are most likely due to socialized gender roles [32, 33]. Thus, our findings that females used more behavioural disengagement, while males used less social support to deal with stress, may reflect gender stereotypes that are reinforced within the medical culture. Accordingly, it could be that male medical students prefer to be independent and not to reach out for social support (e.g., to peers or programs) because doing so might conflict with traditional ideals of what it means to be male (e.g., toughness, independence, and emotional control). Conversely, female medical students may behaviourally disengage because of pressures to demonstrate traditional female traits (e.g., politeness and nurturance), which preclude them from feeling that they can be bold or exert more assertiveness. Indeed, studies suggest that gender-based norms are reinforced in medicine (e.g., via instructor evaluations) and may contribute to distress for female medical students [77]. These are worthy considerations in medical education, given academic stress and stigma around mental health are proven determinants of students’ well-being [23, 52, 78].

### Implications in medical education

Findings from this study may have several important implications in medical education. The first is the need to address gender stereotypes in medicine, that can have potentially detrimental consequences for medical students. For some males, these pressures might mean not seeking external supports and feeling like they need to “tough it out” on their own. For some females, it might mean disengaging or tempering their self-expression, due to a social construction that assertiveness is not a feminine trait. The extent that these gendered constructs may be contributing to additional, unnecessary distress for medical learners is worth exploring.

Another implication is the positive association we found between age and planning, which positively correlated with other adaptive coping strategies (e.g., acceptance, positive reframing, emotional and instrumental support) and negatively correlated with various maladaptive ones (e.g., behavioural disengagement). This suggests that those who are older when they enter into medical school—who likely have prior life experience beyond an educational setting—are potentially better equipped to cope more proactively with stress, such as in clinical settings. If true, providing early support of medical students who are younger and/or who have less experience—for example, in recognizing the benefit of planning ahead (e.g., with study schedules, extra-curricular activities, course rotations, and elective planning)—may help them cope more adaptively with stressors they face, as they progress in their medical education. That said, further research is warranted to assess whether age-related differences in coping are not unique to our institution, but a consistent finding in other medical programs.

Although we did not have a concurrent measure of wellness or distress, findings from this study also suggest that medical students may use a variety of coping strategies that may be viewed as dysfunctional in the main, but in the context of medical school, may in fact be stress protective. For example, our finding that venting emotions (which is often considered maladaptive [10, 15]) positively related to seeking emotional support, instrumental support, acceptance, and humour (which are largely adaptive for well-being [14, 15]) suggests that both male and female medical students may find venting therapeutic. Given the social nature of medical school and that students share the same types of stress, it stands to reason that venting to each other may help them come to terms with stressors they feel are mutually troubling (e.g., performance pressures during courses, exams, or clinical rotations). The weak but significant positive correlation we found between venting and acceptance supports that idea. While it remains unclear how duration of coping strategy (e.g., venting) impacts whether it becomes maladaptive for medical students, creating physical spaces (e.g., lounges) for them to congregate—where they can feel free to vent and decompress—is nevertheless recommended.

Finally, our finding that students in third year reported more use of denial (which is generally considered dysfunctional [14]) than all other years is concerning. We would argue this reflects the uncontrollability of the stressors that third year presents, which can be unfamiliar and disarming for medical students. Although no other coping differences were found between medical students in different years, our analyses did reveal a significant positive correlation between denial and various other maladaptive coping strategies (e.g., substance use, self-blame, and behavioural disengagement). Hence, while we felt reassured that students’ use of denial did show a decrease in fourth year, being aware that denial may be high in third year and that it may pose risks (i.e., in terms of using other maladaptive coping behaviours) may be important for people in positions that support medical students during this time.

As mentioned, a potentially important avenue for achieving this may involve finding ways to facilitate more autonomy for medical learners during their clerkship (e.g., see Neufeld and Malin [79]). Studies suggest that promoting medical learner autonomy may also reduce their perceived stress and increase their ability to be mindful and resilient, which are also key to healthy coping and well-being [76, 80, 81]. Because third year inevitably involves more administrative tasks (i.e., organizing clinical electives and residency applications), which are increasingly being recognized as underrated sources of stress for medical students, we would also echo others’ suggestions to focus on addressing systems-level changes in medical education [52]. Doing so might help to support senior medical students—particularly around career planning and residency preparation—as they move towards graduation and starting residency.

### Limitations & future research

The present study has limitations which may help to guide future research. First, while the response rates were satisfactory for sample representativeness and statistical power, the unequal sample sizes across year subgroups, combined with our reliance on self-report data, both create the potential for response bias. That said, various studies concerning four-year medical programs also point to third year as a highly stressful time for students [48, 82], and demonstrate that medical students (and females in particular) tend to experience more stress and cope less adaptively during this time period [45, 76]. Nonetheless, caution is recommended when interpreting the results from this study, and cohort studies as well as qualitative approaches will be very helpful in enriching these findings and determining their underlying causality.

A second point of mention is that this study relies on a cross-sectional, quantitative (i.e., reductionist) approach and was conducted at a single institution, which limits generalizability and prevents conclusions about temporal patterns of coping (i.e., across years). Thus, while our demographic findings have potential implications in medical education, there is some potential for cohort effects. Additionally, while different gender options (e.g., non-binary gender and transgender) were considered, in retrospect, we recognize that we may not have used the most appropriate approach to measure this. For example, had many students selected ‘Other’ for gender, it would have made drawing conclusions about gender-based differences in coping more complex to disentangle. Future studies would therefore benefit by including a more inclusive range of options to explore these matters further.

Finally, we focused not on coping as a predictor of mental health outcomes, but on how frequently medical students used specific coping strategies and how they are influenced by their gender and year of study. While our results suggest that these demographic antecedents are indeed important determinants of medical students’ coping responses, further studies are needed to confirm these findings. Others may also wish to examine other factors that could influence coping (e.g., psychological diagnoses, prior work experience, ethnicity, marital and socioeconomic status, and career-related goals), as well as include concurrent measures of academic performance and/or positive (e.g., subjective well-being) and negative (e.g., perceived stress) mental health. This might help to capture not only the types of coping strategies medical students use during medical school, but how those strategies relate to their health and functioning.

## Conclusion

This study contributes to a growing body of research on stress and coping among medical students. Our findings intimate that female medical students may engage in more behavioural disengagement, while males may utilize less emotional and instrumental support. Our results also suggest that third year learning environments may potentiate more maladaptive coping strategies, such as denial, compared to those in other years. Thus, while further research is needed to validate these findings, the presents study adds a new perspective to the debate on how medical students cope with stress in medical school and what the role of gender and year of study are in that relationship. We hope this study provides an impetus for medical educators to create supports that address gendered constructs of coping, as well as learning environment interventions in third year, that foster the well-being of medical students.

## Data Availability

The dataset used and/or analyzed during the current study are available from the corresponding author on reasonable request.
